# Automated Model Inference for Gaussian Processes: An Overview of State-of-the-Art Methods and Algorithms

**DOI:** 10.1007/s42979-022-01186-x

**Published:** 2022-05-21

**Authors:** Fabian Berns, Jan Hüwel, Christian Beecks

**Affiliations:** 1 University of Hagen, Hagen, Germany; 2 Fraunhofer Institute for Applied Information Technology FIT, Sankt Augustin, Germany

**Keywords:** Gaussian processes, Machine learning, Probabilistic machine learning

## Abstract

Gaussian process models (GPMs) are widely regarded as a prominent tool for learning statistical data models that enable interpolation, regression, and classification. These models are typically instantiated by a Gaussian Process with a zero-mean function and a radial basis covariance function. While these default instantiations yield acceptable analytical quality in terms of model accuracy, GPM inference algorithms automatically search for an application-specific model fitting a particular dataset. State-of-the-art methods for automated inference of GPMs are searching the space of possible models in a rather intricate way and thus result in super-quadratic computation time complexity for model selection and evaluation. Since these properties only enable processing small datasets with low statistical versatility, various methods and algorithms using global as well as local approximations have been proposed for efficient inference of large-scale GPMs. While the latter approximation relies on representing data via local sub-models, global approaches capture data’s inherent characteristics by means of an educated sample. In this paper, we investigate the current state-of-the-art in automated model inference for Gaussian processes and outline strengths and shortcomings of the respective approaches. A performance analysis backs our theoretical findings and provides further empirical evidence. It indicates that approximated inference algorithms, especially locally approximating ones, deliver superior runtime performance, while maintaining the quality level of those using non-approximative Gaussian processes.

## Introduction

Applying Gaussian Process Models (GPMs) for interpolation [26, 38], regression [14, 45], and classification [19, 26] necessitates to instantiate the underlying Gaussian Process by a covariance function and a mean function. While the latter is typically instantiated by a constant, zero-mean function, the covariance function is modelled either by (i) a *general-purpose kernel* [50], (ii) a *domain-specific kernel* that is individually tailored to a specific application by a domain expert [1, 36, 50], or (iii) a *composite kernel* that is computed automatically by means of a GPM inference process [14, 28] in order to decompose the statistical characteristics underlying the data into multiple sub-models. Although the first option (i) produces GPMs of sufficiently high model accuracy, both (ii) and (iii) encapsulate data’s peculiarities and versatilities in a more detailed manner due to their data-informed approaches. Making use of domain-specific kernels requires laborious fine-tuning and extensive expert knowledge. Employing an automated, domain-agnostic GPM inference process, on the other hand, produces expressive composite kernels for diverse data analytical tasks without requiring any human interference.

Exhaustive automated GPM inference algorithms apply an open-ended greedy search in the space of all feasible composite covariance functions in order to determine an optimal instantiation of the underlying Gaussian Process [14, 25, 28, 43]. The resulting inextricable GPM needs to account for all different kinds of statistical peculiarities of the underlying data and thus potentially lacks expressiveness with regards to local phenomena. Additionally, evaluating a GPM by usual measures such as the likelihood [31] function entails calculations of cubic computation time complexity, which limits the application of GPM inference algorithms to small-to-moderate data collections [25]. Therefore, locally approximated algorithms [6, 8, 9] concurrently initiate multiple local inference procedures on dynamically *partitioned* data, which enable efficient inference of GPMs for dataset on the scale of millions of records.

In this paper, we provide an overview of the current state of the art of *automated Gaussian Process Model inference* [6–9, 14, 25, 28, 43] presenting both an empirical perspective as well as the theoretical background information. First, we present related work and background information on Gaussian Processes in “Background and related work”. Secondly, we provide an overview of current state-of-the-art algorithms for Automated GPM Inference in Sect. "Automated model inference for Gaussian processes". Thirdly, their individual performance with regards to several uni- and multidimensional benchmark datasets (i) indicating the performance of state-of-the-art GPM inference algorithms and (ii) highlighting their individual strengths and shortcomings is presented in "Evaluation". Finally, we conclude our paper in "Conclusion".

## Background and Related Work

### Gaussian Process

Gaussian Processes are non-parametric probabilistic kernel machines [36] that offer a statistically tractable and interpretable data representation [19, 28]. They have a wide range of possible applications in data analysis, including regression for uni- and multivariate data [42, 45], nearest neighbour analysis [13], interpolation [26], classification [42] and clustering [24]. They are also used for information retrieval in biology [49] and image processing [29]. In recent works, Gaussian Processes have been used for forecasting the spread of COVID-19 [4]. They are applied in 3D-animation [44] to model the influence of emotions on movements, and in traffic simulations [2] to predict human behavior. Overall, they are considered a prominent method in the field of probabilistic machine learning [15].

Gaussian Processes [36] are categorized as stochastic processes over random variables $\{f(x) \, | \, x \in \mathcal {X}\}$, indexed by a set $\mathcal {X}$, where every subset of random variables follows a multivariate normal distribution. The distribution of a Gaussian Process is the joint distribution of all of these random variables and it is thus a probability distribution over the space of functions $\{f\, | \,f:\mathcal {X} \rightarrow \mathbb {R} \}$. A Gaussian Process is formalized as follows:

1 $$\begin{aligned} f(\cdot ) \sim GP\left( m(\cdot ), k(\cdot ,\cdot )\right) \end{aligned}$$

where the mean function $m: \mathcal {X} \rightarrow \mathbb {R}$ and the covariance function $k : \mathcal {X} \times \mathcal {X} \rightarrow \mathbb {R}$ are defined $\forall x \in \mathcal {X}$ as follows:

2 $$\begin{aligned} m(x)&= \mathbb {E}\left[ f(x)\right] \end{aligned}$$

3 $$\begin{aligned} k(x, x')&= \mathbb {E}\left[ \left( f(x)-m(x)\right) \cdot \left( f(x')-m(x')\right) \right] \end{aligned}$$

Given a finite dataset $D=\{X, y\}$ with $X=\{x_i \, | \, x_i \in \mathcal {X} \wedge 1 \le i \le n\}$ representing the underlying input data (such as timestamps) and $y \in \mathbb {R}^{n}$ representing the target data values, such as sensor values or other complex measurements, hyperparameters $\theta$ of the mean and covariance functions are optimized by maximizing the log marginal likelihood [36] of the Gaussian Process, which is defined as follows:

4 $$\begin{aligned} {\mathcal {L}}(m, k, \theta \, | \,D)= & {} -\frac{1}{2} \cdot \left[ (y-\mu )^{T} \varSigma ^{-1} (y-\mu ) \right. \nonumber \\&\left. + \log \det (\varSigma ) + n \log (2\pi )\right] \end{aligned}$$

As can be seen in Eq. (4), the log marginal likelihood of a Gaussian Process for a given dataset *D* of *n* records relies on mean vector $\mu \in \mathbb {R}^{n}$, and covariance matrix $\varSigma \in \mathbb {R}^{n \times n}$ which are defined as $\mu [i] = m(x_i)$, and $\varSigma [i,j] = k(x_i,x_j)$ for $1\le i,j\le n$, respectively. We use the short-hand notation $GP_\theta$ to denote a GPM whose underlying mean function *m* and covariance function *k* are parameterized via hyperparameters $\theta$ (cf. [10, 12]). Commonly, the covariance function is structured via multiple compositional covariance functions in order to obtain the resulting compositional kernel-based covariance function [8].

Since computation time complexity of evaluating and applying an exact Gaussian process lies in $\mathcal {O}(n^3)$ [19], different approximation techniques have been developed [41]. In the following two subsections, we outline the two major categories of Gaussian process approximations, i.e. local and global approximation, and present prominent representatives for each category.

### Global Approximations

One prominent type of approximation used to speed up Gaussian Process evaluation and application is the group of global approximations [27, 41]. Instead of considering every single data record of a dataset of interest, these approximations focus on a (ideally representative) sample [27]. The family of Subset-of-Data (SoD) approaches [17] only use $m \ll n$ data points for training, reducing complexity to $\mathcal {O}(m^3)$. While these SoD approaches leave the general framework of a Gaussian Process unchanged and thus offer an easy solution to the problem of runtime complexity, they often suffer from an “overconfident prediction variance” [27] in comparison to other global approaches such as sparse approximations [16]. Although the latter still utilize a set of inducing points considerable smaller than the training set, they are better suited to make predictions with regards to the *whole* training set [27].

These approximations include the Nyström-approximation [36], a low-rank approximation of the covariance matrix ($\mathcal {O}(n m^2)$), which became quite prominent in an optimized form either called Sparse Pseudo-input Gaussian Process (SPGP) [40] or Fully Independent Training Conditional (FITC) [5]. Furthermore, Titsias [45] introduced Variational Free Energy (VFE), which also improves on the basic Nyström-approximation via a regularization term for the likelihood. Orthogonal to these improvements, Wilson and Nickisch [51] introduce the Structured Kernel Interpolation (SKI) method to further speed up these kinds of matrix approximations by linearly interpolating the full covariance matrix based on a small set of inducing data points *m*.

In general, these global approximations are able to capture global patterns, but lack capabilities to capture local ones due to their sampling nature [25, 27]. Thus, globally approximated Gaussian Processes focus on the expressive capacity of their respective sample rather than the full dataset.

### Local Approximations

Local approximations are the other major group of Gaussian Process approximations and utilize *local experts*, i.e. distinct Gaussian Process sub-models for data segments. They are organized in three categories. While mixture-of-experts (MoE) [32, 53] and product-of-experts (PoE) approaches [20] try to smooth out the transition between segments and experts, only strict, thus non-smoothing approaches (also called Naïve Local Experts (NLE) [27]) can fully exploit the algorithmic advantages of local approximations [37, 41]. Therefore, current state-of-the-art GPM inference algorithms focus on the latter kind, which are simply called *local approximations* throughout this paper for the sake of simplicity.

Liu et al. [27] as well as Rivera and Burnaev [37] highlight these approximations as a key possibility to reduce complexity of common GPM evaluations based on likelihood measures. Instead of inferring an approximate GPM based on a data subset (cf. *global* approximations), they construct a holistic GPM from locally-specialized models trained on non-overlapping partitions of the data. Thus, the covariance matrix of the holistic GPM is composed of the respective matrices of its local sub-models [27]. This divide- &-conquer approach accelerates calculation of log marginal likelihood, since the resulting covariance matrix is a block diagonal matrix [37], whose inverse and determinant can be computed efficiently [34]. Rivera and Burnaev [37] emphasize that *local approximations* allow to “model rapidly-varying functions with small correlations” in contrast to low-rank matrix approximations.

Embedding the concept of *local approximations* into GPM inference algorithms requires a globally partitioning covariance function *K*. We use an indicator functions (also called *Heaviside step function* [22]) to concatenate independent sub-models into one coherent, partitioned GPM. This leads us to the definition of a partitioning covariance function *K*. We employ a predefined set of disjoint partitions $\mathcal {P} = \{X_i, y_i\}_{i=1}^a$ to partition *K* into *a* independent sub-models $k_i$:

5 $$\begin{aligned} K(x,x' | \{k_i\}_{i=1}^a, \mathcal {P}) = \sum _{i=1}^{a}{k_i(x,x') \cdot \mathbbm {1}_{ x \in X_i }(x) \cdot \mathbbm {1}_{ x' \in X_i }(x')} \end{aligned}$$

The parameter $a \in \mathbb {N}$ defines the number of sub-models $k_i: \mathcal {X} \times \mathcal {X} \rightarrow \mathbb {R}$, where each sub-model $k_i$ can be thought of as a local covariance function modeling the restricted domain $X_i \subseteq \mathcal {X}$. The usage of indicator functions (thus having disjoint data segments) produces a block diagonal covariance matrix [30], which facilitates independently and thus efficiently searching the most likely local covariance function (i.e. sub-model) per data segment.

To mathematically show the implication of aforementioned independence, we have to investigate the internal structure of the corresponding log marginal likelihood function $\mathcal {L}(m, k, \theta \, | \,D)$. To this end, we assume the covariance matrix $\varSigma \in \mathbb {R}^{n\times n}$ (cf. Eq. 4) to be a block diagonal matrix. That is, it holds that $\varSigma = diag(\varSigma _1, ..., \varSigma _m)$, where each matrix $\varSigma _i \in \mathbb {R}^{n_i\times n_i}$ for $1 \le i \le m$ is of individual arbitrary size $n_i \in \mathbb {N}$ such that $\sum _i{n_i} = n$. Furthermore, let $(\mathcal {I}_1, ..., \mathcal {I}_m)$ denote the partitioning of index set $\{1, ..., n\} \subseteq \mathbb {N}$ according to the given blocks, i.e. for a submatrix of $\varSigma$ over index set $\mathcal {I}_i$ the following holds: $\varSigma _{\mathcal {I}_i, \mathcal {I}_i} = \varSigma _i \forall i \in [1,m]$. Given this notation, one can show (i) that the quadratic form $(y-\mu )^T \varSigma ^{-1} (y-\mu )$ of the log marginal likelihood function $\mathcal {L}(m, k, \theta \, | \,D)$ is equivalent to $\sum _{i=1}^{a}{(\tilde{y}_i-\tilde{\mu }_i)^T \varSigma _i^{-1} (\tilde{y}_i-\tilde{\mu }_i)}$, where $\tilde{y}_i = (y_i)_{I\in \mathcal {I}_i} \in \mathbb {R}^{n_i}$ and $\tilde{\mu }_i = (\mu _i)_{I\in \mathcal {I}_i} \in \mathbb {R}^{n_i}$ are defined according to the block diagonal structure and (ii) that the logarithmic determinant can be decomposed as follows: $\log \det (\varSigma ) =\log \prod _{i=1}^{a}{\det (\varSigma _i)} = \sum _{i=1}^{a}{\log \det (\varSigma _i)}$. By making use of the aforementioned algebraic simplifications, we can simplify the calculation of the log marginal likelihood function as shown below:

6 $$\begin{aligned} \begin{aligned}&\mathcal {L}(m, k, \theta \, | \, D)\\&\quad =-\frac{1}{2} \cdot \left[ (y-\mu )^T \varSigma ^{-1} (y-\mu ) + \log \det (\varSigma ) + n \log (2\pi )\right] \\&\quad =-\frac{1}{2} \cdot \left[ \sum _{i=1}^{a}{(\tilde{y}_i-\tilde{\mu }_i)^T \varSigma _i^{-1} (\tilde{y}_i-\tilde{\mu }_i)}\right. \\&\qquad \left. + \sum _{i=1}^{a}{\log \det (\varSigma _i)} + \sum _{i=1}^{a}{n_i \cdot \log (2 \pi )}\right] \\&\quad =\sum _{i=1}^{a} -\frac{1}{2} \cdot \left[ (\tilde{y}_i-\tilde{\mu }_i)^T \varSigma _i^{-1} (\tilde{y}_i-\tilde{\mu }_i) \right. \\&\qquad \left. + \log \det (\varSigma _i) + n_i \log (2\pi )\right] \\&\quad =\sum _{i=1}^{a}{\mathcal {L}(m, k_i, \theta \, | \, \mathcal {P})}\\ \end{aligned} \end{aligned}$$

As shown above, the equivalence $\mathcal {L}(m, k, \theta \, | \,D) = \sum _{i=1}^{a}{\mathcal {L}(m, k_i, \theta \, | \,\mathcal {P})}$ affects the computation of a GPM by means of *K* for a given data set *D* and its non-overlapping partitions $\mathcal {P}$. Instead of jointly optimizing a holistic statistical model fitting the entire data set *D*, we are able to independently optimize individual sub-models $k_i$. This will not only increase the expressiveness of the overall GPM, but also reduce the computation time required for calculating such a model.

## Automated Model Inference for Gaussian Processes

In general, inference is regarded as a process that addresses the uncertainty involved in choosing an apt instantiation of a variable facing certain observations. While Gaussian Process inference focuses mainly on hyperparameter optimization [19] (and if applicable finding optimal inducing points and inputs [39]) for a *predefined* model comprising specific covariance and mean function, inference of Gaussian Process *models* also takes the uncertainty of the latter constituents and their structure into account to infer the whole model. For this purpose, the mean function of the Gaussian Process is commonly instantiated by a constant zero function [14, 36], so as to correspond to an additional data normalization step. The second constituent, i.e. covariance function *k*, is algorithmically composed via operators implementing addition and multiplication among different (composed) base kernels $b \in \mathcal {B}$. Prominent base kernels include the linear kernel, Radial Basis Function (RBF) kernel, and periodic kernel, which are able to capture for instance smooth, jagged, and periodic behavior [14].

In general, we define automatic GPM inference as follows (cf. [6]):

### Definition 1

(*Automatic GPM inference*) Automatic GPM inference means to search a search space of GPMs $\mathcal {G}$ for an optimal GPM $GP_\theta ^* \in \mathcal {G}$, which is not outperformed by any other GPM $GP_\theta \in \mathcal {G}$ in terms of a predefined quality measure, here exemplarily instantiated by the likelihood $\mathcal {L}(m, k, \theta \, | \, D)$:

$$\begin{aligned} GP_\theta ^* \in {{\,\mathrm{arg\,max}\,}}_{GP_\theta (m, k) \in \mathcal {G}}{\mathcal {L}(m, k, \theta \, | \, D)} \end{aligned}$$

In this section, we outline state-of-the-art, automatic GPM inference algorithms organized into the categories of exhaustive (cf. "Exhaustive model inference") and partitioned (cf. "Partitioned model inference") inference algorithms. While the latter consists of those employing local approximations and is thus suited for big data, the first encompasses approaches producing one universal, inextricable GPM for the whole dataset.

### Exhaustive Model Inference

Exhaustive inference algorithms, such as Compositional Kernel Search (CKS) [14], Automatic Bayesian Covariance Discovery (ABCD) [28, 43], and Scalable Kernel Composition (SKC) [25], apply an open-ended, greedy search in the space of all feasible kernel combinations to progressively compute a GPM fitting the entire dataset *D*, respectively $y \in D$. The CKS algorithm [14] follows a simple iterative procedure, which aims to improve the best covariance function *k* inferred in one iteration over the course of the next one. Therefore, this strategy produces a set of candidate covariance functions $\mathcal {C}_k$ based on the previous best covariance function *k*. In doing so, a new candidate is created by extending any subexpression of *k* by means of a further base kernel $b \in \mathcal {B}$ [14]. We express this candidate generation via the following term, abstracted for the sake of comprehensibility:

7 $$\begin{aligned} \mathcal {C}_k = \left\{ k \oslash b \, | \, b \in \mathcal {B} \wedge \oslash \in \{ +, \times \}\right\} \end{aligned}$$

ABCD extends that candidate set with regards to a sigmoid change point operator $\mathbf ]\![$ [43], to locally restrict the effect of covariance functions [28]:

8 $$\begin{aligned} \mathcal {C}_k = \left\{ k \oslash b \, | \, b \in \mathcal {B} \wedge \oslash \in \{ +, \times , \mathbf ]\![ \} \right\} \end{aligned}$$

Algorithm 1 summarizes steps taken by exhaustive inference algorithms, where candidate GPMs of increasing complexity are evaluated in an iterative procedure until either further complexity is not improving model quality or a complexity bound $c(k_{best}) < c_{max}$ is met. Usually, complexity bound $c(\cdot )$ represents an upper bound for the amount of base kernels constituting covariance function $k_{best}$. CKS and ABCD only differ in their candidate set $\mathcal {C}_k$ (cf. Eqs. 7 and 8). Starting with a set of base kernels $\mathcal {B}$, the candidates of every following iteration are generated via expanding upon the best candidate of the previous iteration. Moreover, a new candidate is also generated for every *replacement* of a base kernel *b* with another one $b'$.
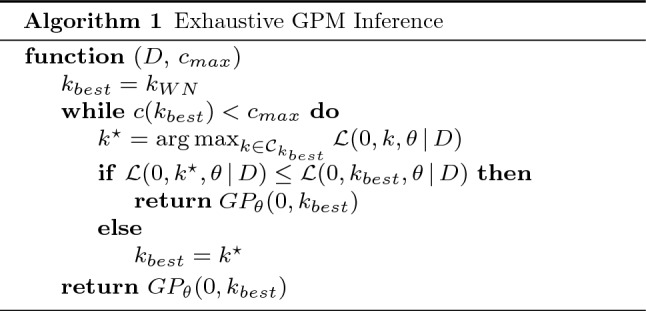


The performance of those given automated GPM inference algorithms is impeded by two major bottlenecks. Evaluating *every* candidate conceived by expanding on *any* subexpression of the current best covariance function $k_{best}$ poses one of these bottlenecks. Thus, reducing the set of candidates per iteration to cover just the most promising candidates improves on the performance of the respective algorithms, presumably without affecting model quality. Moreover, assessing model quality in terms of log marginal likelihood epitomizes another bottleneck of current algorithms, which is intrinsic to the framework of Gaussian Processes itself [19]. The cubic runtime complexity of that basic measure inhibits analysis of large-scale datasets (cf. [25]). Kim and Teh [25] present SKC as a way to overcome at least the latter one of those bottlenecks. The algorithm improves the efficiency of the kernel search process by accelerating CKS using VFE [45] for hyperparameter optimization and a custom upper bound on $\mathcal {L}$ for determining $k_{best}$ [25]. These approximations enable SKC to infer GPMs for datasets of up to 100,000 records in a reasonable amount of time according to Kim and Teh [25]. Furthermore, SKC uses a candidate generation strategy, which produces fewer candidates. In particular, this strategy only considers candidates generated by expanding the full $k_{best}$ by further added base kernels $b \in \mathcal {B}$ instead of expanding every subexpression [25].^1^

To summarize, state-of-the-art exhaustive, automated GPM inference algorithms are well applicable to small datasets but are not suited for the analysis of large-scale datasets beyond 100,000 records due to their cubic computation time complexity as well as due to their large candidate sets, which need to be evaluated during the search procedure [25].

### Partitioned Model Inference

In this section, we outline partitioned GPM inference algorithms making use of the efficiency improvement of locally approximated covariance function *K* (cf. Eq. 5) and a more rigid candidate selection. Although differing in their algorithmic design, all partitioned inference approaches use the same formalization of candidate set $\mathcal {C}_k$ for inferring local sub-models later concatenated by *K* [6–9]. They restrict the candidate set to those covariance functions complying with the sum-of-products form and subsequently reduce redundancy in the candidate set [8]:

9 $$\begin{aligned} \begin{aligned} \mathcal {C}_k =&\left\{ k \oslash b \,|\, b \in \mathcal {B}, \oslash \in \{ +, \times \}, (k \oslash b) \in \mathcal {S} \right\} \\ \mathcal {S} =&\left\{ \sum {\prod {b}} \,|\, b \in \mathcal {B} \right\} \end{aligned} \end{aligned}$$

The Large-Scale Automatic Retrieval of GPMs (LARGe)^2^ algorithm [6, 7] initiates multiple local kernel searches on dynamically partitioned data. Subsequently, LARGe concatenates the resulting sub-models, i.e. each independent composite kernel-based covariance function $k_i$, into a joint large-scale GPM by means of *K* (cf. Eq. 5) and a zero-mean function. Prior to the computation of these independent sub-models, a global partitioning needs to be determined in advance. We use clustering algorithms [52] for multi-dimensional data and change point detection mechanisms [3, 46] for uni-dimensional input data. Besides $\mathcal {P}$, the given dataset $D=\{X,y\}$ (cf. "Gaussian process") and complexity parameter $c_{\rm max}$, restricting sub-model size, are given as parameters [6].

Algorithm 2 illustrates the LARGe algorithm. Without loss of generality we initiate the sub-models $k_i \in K$ for every data partition $\mathcal {P}$ via a white noise kernel $k_{WN}$. The set $\mathcal {I}$ encompasses the indices of all non-final sub-models $k_i \in K$. The following while-loop is executed until this set of indices is empty. During every iteration the segment $S_i$ with the lowest model quality by means of log marginal likelihood $\mathcal {L}$ is selected, candidate covariance functions $\mathcal {C}_{S_i}$ for that segment are found using the predefined candidate generation strategy and the best performing covariance function $k^{\star }$ is determined. This mechanism enables to return a preliminary GPM early on in the process [6].^3^ If that covariance function $k^{\star }$ reaches maximum complexity $c_{max}$ in terms of number of involved base kernels or delivers equal quality w.r.t. the previous best expression $S_i$, it is considered final and thus removed from $\mathcal {I}$. Finally, a GPM utilizing a *K*-based (cf. Eq. 5) concatenation of sub-models $k_i \in K$ and zero-mean is returned [6, 7].
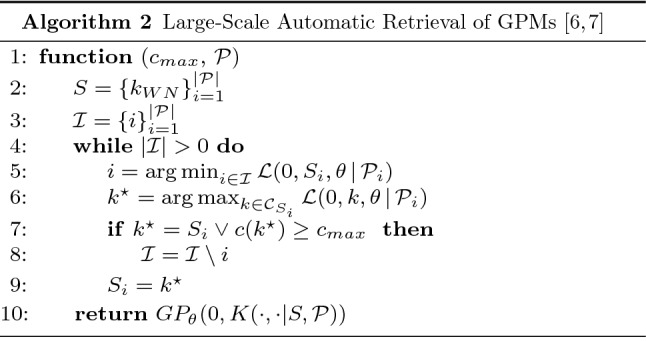


The Concatenated Composite Covariance Search (3CS) [9] algorithm follows similar steps for finding a locally approximated GPM for a given dataset *D* given a complexity constraint $c_{max}$^4^, but is based on a sequential procedure and a Gaussian-process-based change point detection mechanism [9]. The latter uses the partitioning covariance function *K* and two partitions resulting from a single change point $\tau$ separating two white noise kernels $k_{WN}$ to find an optimal change point $\tau ^*$ in the current data partition of interest. 3CS uses a sliding window $D_i$ to describe this data partition of size *w*, searches for an apt change point and employs *exhaustive search* with the adjusted candidate set defined in Equation 9 to find an optimal local model $k^\star$ [8, 9].
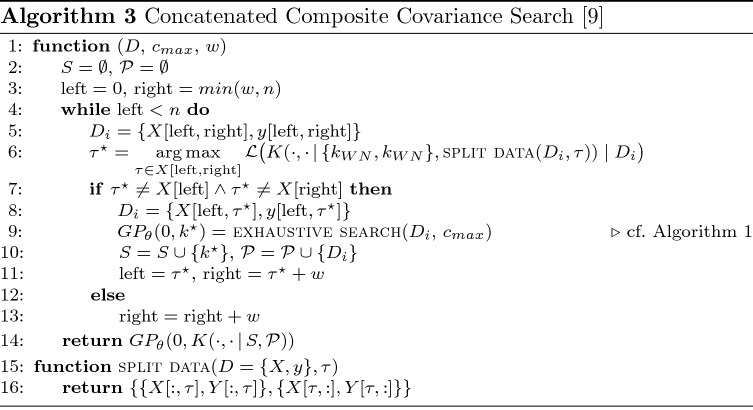


Finally, Algorithm 4 illustrates Lineage GPM Inference (LGI) [8], which aims to overcome one of the major issues of local approximations, i.e. very large partitions. Since every local model of a locally approximated GPM is virtually an exact GPM on its own, large partitions cause the same runtime issues faced by non-approximative Gaussian Processes [8]. Thus, LGI was proposed to overcome that problem by employing local *and global* approximations and alternating between both over the course of its recursive procedure. At first, the recursive search is started with regards to the whole dataset *D*, complexity constraint $c_{max}$, and a white-noise kernel as initial model. Utilizing a global approximation $\varOmega$ an improved model $k^\star$ for the current data partition of interest $\hat{D}$ is found. If an improvement with regards to the previous model $\hat{k}$ is not possible, the previous model is returned. Otherwise, $\hat{D}$ is partitioned and recursive search is started again for all resulting partitions. Eventually, LGI is designed to find “increasingly particularized sub-models for partitions of further shrinking size” [8].
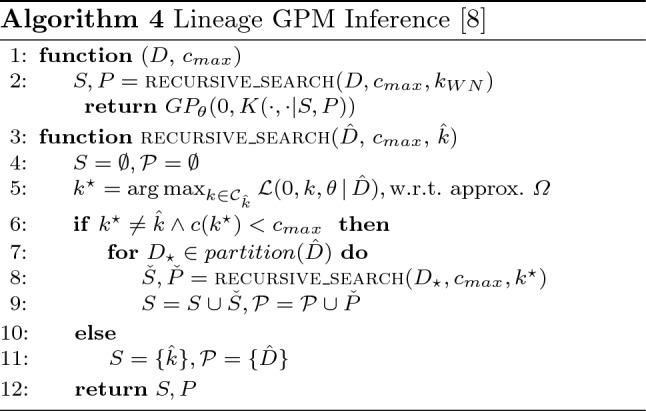

**Table 1:** Major distinctive, conceptual properties of state-of-the-art, automated GPM inference algorithms

Algorithm	Global approx.	Local approx.	Multidim. input *X*	Max. |*X*|
CKS			$\checkmark$	$\sim 10k$ [14, 25]
ABCD				$\sim 10k$ [25, 28]
SKC	$\checkmark$		$\checkmark$	$\sim 100k$ [25]
LARGe		$\checkmark$	$\checkmark$	$> 2M$ [6, 7]
3CS		$\checkmark$		$> 2M$ [9]
LGI	$\checkmark$	$\checkmark$	$\checkmark$	$> 2M$ [8]

In this section, we have outlined the state-of-the-art, automated GPM inference algorithms, both using exact Gaussian Processes as well as approximative ones. Their respective commonalities and differences with regards to utilized approximations, ability to analyze multi-dimensional data, and largest dataset size processable in a reasonable amount of time according to the literature are summarized by Table 1. Their individual merits and weaknesses are further empirically analyzed in the following section.

## Evaluation

In this section, we investigate the performance of the outlined GPM inference algorithms in terms of runtime efficiency and accuracy. In order to ensure comparability with existing and prospective algorithms, we base our experiments on publicly available datasets, which were used in the majority of other papers proposing GPM inference algorithms [6–9, 14, 25, 28] (cf. Table 2). Kim and Teh [25] note that datasets larger than a few thousand points are *“far beyond the scope of [...] GP optimisation in CKS”* and consequently beyond the scope of ABCD as well. Therefore, datasets 1–6 are rather small. Especially for scalability analysis, we include five larger datasets (datasets 7–11 in Table 2). Besides covering datasets of different sizes, the given collection of datasets also includes different application domains and dimensionalities.

**Table 2:** Used benchmark datasets

#	Dataset	Size	Dimensions
1	Airline [14, 28]	144	2
2	Hardware [23]	209	7
3	Solar Irradiance [14, 28]	391	2
4	AutoMPG [35]	392	8
5	Mauna Loa [14, 28]	702	2
6	Energy [47]	768	9
7	SML System^a^ [54]	4,137	24
8	Power Plant^b^ [48]	9,568	5
9	GEFCom^c^ [21]	38,064	2
10	Jena Weather^d^ [33]	420,551	15
11	Household Energy^e^ [18]	2,075,259	9

^a^Dimension “Carbon dioxide in ppm (room)” was used as uni-dimensional target *Y*

^b^ Dimension “Electrical power output ($P_E$)” was used as uni-dimensional target *Y*

^c^ We chose one of the 20 utility zones (i.e. the first one) as the informational content among zones may be considered equivalent [21]

^d^Dimension “Air_Temperature” was used as uni-dimensional target *Y*. We are using the recognized interval of this continuously gathered dataset from 2009-01-01 to 2016-12-31 (cf. [11])

^e^Dimension “Global_active_power” was used as uni-dimensional target *Y*

All experiments are executed on a multi-core machine with 3.9 GHz CPU and 128 GB main memory. The latter capability accommodates for the high storage complexity, i.e. $\mathcal {O}(n^2)$, cf. [19], of non-approximate Gaussian Process Inference by means of CKS and ABCD. All algorithms were implemented using Python 3.9 and Tensorflow 2.7 (no GPU acceleration enabled). Since commonly likelihood $\mathcal {L}$ is just used for optimizing models and Root Mean Squared Error (RMSE) for assessing model quality [6, 14], we will adopt that procedure as well. 90% of the respective data is used for training, while the remaining 10% are kept aside for determining test accuracy. To produce reliable results every single run of an algorithm is repeated with five different train-test-splits. Thus, Tables 4 and 3 report on the median metric across these five runs. To thoroughly evaluate every candidate GPM, their hyperparameter optimization is repeated ten times with different hyperparameter initializations (i.e. random restarts [25]).

Since the given six algorithms vary largely with regards to their parallelization capacities, we do not use multi-processing to ensure comparability. Still, we enable Tensorflow to internally parallelize mathematical operations (e.g. matrix inversion and matrix multiplication) using up to eight threads. We use complexity constraint $c_{max}= 4$ for all algorithms, Bottom-Up Segmentation for partitioning uni-dimensional data and k-Means for partitioning multi-dimensional data. Furthermore, LGI uses a SoD approach as global approximation $\varOmega$ (cf. [6–9, 14, 25, 28]).

**Fig. 1:**
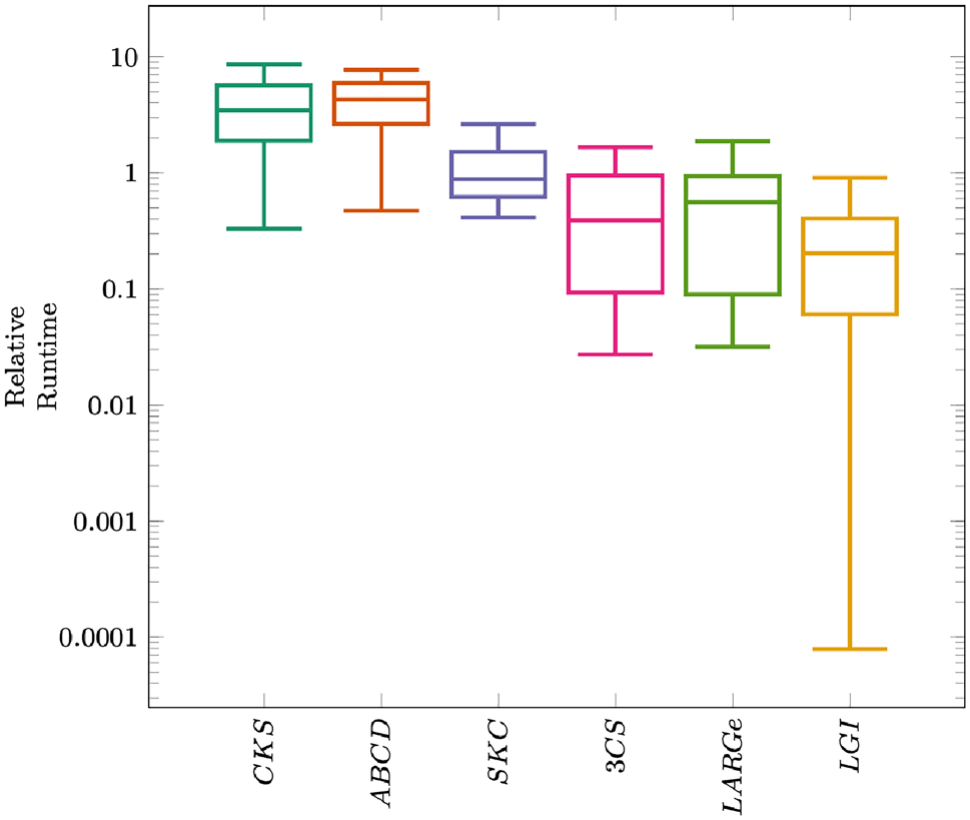
Distribution of relative runtime for different automatic GPM inference algorithms

Figure 1,^5^ summarizes GPM inference runtime for all six algorithms. It becomes apparent that non-approximative methods, i.e. CKS and ABCD, as well as the solely globally approximating method, i.e. SKC, are less efficient in terms of runtime than the remaining locally approximating ones. Therefore, the execution of these less efficient algorithms was omitted for larger datasets (datasets 9–11). Furthermore, LGI is the most runtime-efficient algorithm among the partitioned model inference procedures, while the other two approaches are apparently less efficient. Table 3 further details the efficiency of all six algorithms by stating their runtime with regards to the different considered datasets. Since ABCD and 3CS solely rely on change points for partitioning data, they are only evaluated for uni-dimensional input data.

**Fig. 2:**
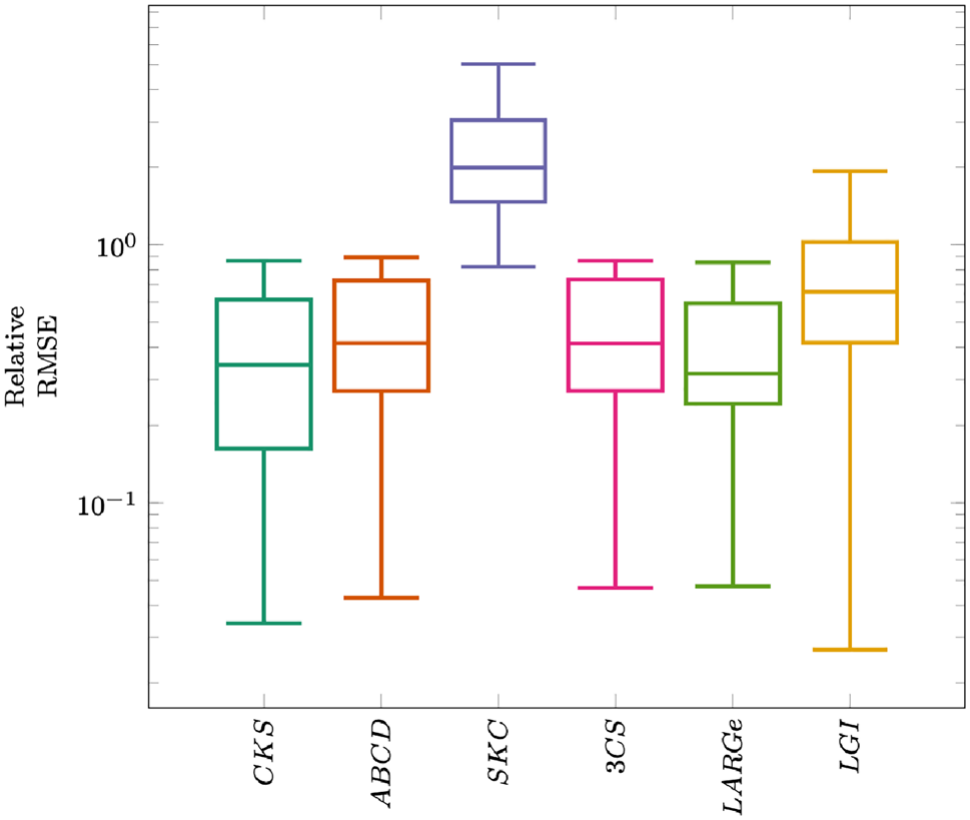
Distribution of model error in terms of RMSE per model resulting from different automatic GPM inference algorithms

To put the findings with regards to runtime efficiency into perspective, Fig. 2,^6^ illustrates the distribution of model accuracy per model inference approach. Despite substantial advantages in runtime efficiency, the models produced by partitioned automatic GPM inference algorithms are able to maintain the level of quality of those models produced by non-approximative algorithms. LGI’s gains in runtime performance come at the cost of a slight reduction in model quality when compared to other partitioned algorithms. Table 4 further details these findings.

**Table 3:** Median efficiency of different automatic GPM inference algorithms in terms of runtime (Format HH:MM:SS)

Dataset	CKS	ABCD	SKC	3CS	LARGe	LGI
**Uni-dim.**						
Airline	00:00:41	00:00:55	00:00:26	00:00:14	00:00:13	00:00:02
Solar Irr.	00:01:14	00:00:46	00:00:16	00:00:11	00:00:25	00:00:09
Mauna L.	00:01:15	00:03:43	00:00:45	00:01:07	00:00:34	00:00:25
SML S.	03:51:53	04:56:29	00:35:20	00:04:00	00:04:50	00:01:59
Power Pl.	36:16:02	46:34:21	05:38:25	00:14:07	00:16:55	00:06:00
GEFCom	–	–	–	00:49:23	00:42:09	00:12:49
Jena W.	–	–	–	07:43:30	05:35:16	04:51:56
House. En.	–	–	–	37:17:50	65:34:23	20:26:09
**Multi-dim.**						
Hardware	00:00:06	–	00:00:25	–	00:00:16	00:00:02
Auto MPG	00:01:35	–	00:00:28	–	00:00:34	00:00:24
Energy	00:05:30	–	00:01:25	–	00:01:00	00:00:42
SML S.	08:14:34	–	00:56:45	–	00:06:16	00:05:59
Power Pl.	26:35:09	–	08:49:59	–	00:12:00	00:07:41
Jena W.	–	–	–	–	06:37:40	14:54:04
House. En.	–	–	–	–	25:35:26	12:01:54

**Table 4:** Median accuracy of models resulting from different automatic GPM inference algorithms in terms RMSE

Dataset	CKS	ABCD	SKC	3CS	LARGe	LGI
**Uni-dim.**						
Airline	0.0438	0.0439	0.3350	0.0438	0.0438	0.0565
Solar Irr.	0.0877	0.0892	0.2689	0.0887	0.1133	0.1201
Mauna L.	0.0030	0.0034	0.4901	0.0032	0.0033	0.0042
SML S.	0.0090	0.0089	0.0591	0.0089	0.0090	0.0273
Power Pl.	0.2240	0.2240	0.2878	0.2252	0.2257	0.2460
GEFCom	–	–	–	0.0093	0.0094	0.0246
Jena W.	–	–	–	0.0024	0.0023	0.0035
House. En.	–	–	–	0.0185	0.0186	0.0221
**Multi-dim.**						
Hardware	0.0352	–	0.0848	–	0.0066	0.0303
Auto MPG	0.1015	–	0.3971	–	0.1025	0.1239
Energy	0.0206	–	0.4846	–	0.0360	0.0701
SML S.	0.0121	–	0.0646	–	0.0191	0.0549
Power Pl.	0.0615	–	0.4980	–	0.0555	0.0833
Jena W.	–	–	–	–	0.0007	0.0001
House. En.	–	–	–	–	0.0032	0.0032

To conclude, we have evaluated the performance of six state-of-the-art GPM inference algorithms on various uni-dimensional and multi-dimensional benchmark databases. Our performance study indicates that partitioned approaches for GPM inference provide higher performance on average in terms of runtime efficiency, while maintaining the level of quality delivered by non-approximative algorithms.

## Conclusion

In this paper, we have investigated automated GPM inference algorithms. In doing so, we first exposed the mathematical and statistical background of Gaussian Processes and current approaches to apply them in an approximate manner. Based on that theoretical background, we outlined state-of-the-art inference algorithms and highlighted the respective differences and commonalities.

Furthermore, we evaluated these algorithms from a practical perspective. Our performance evaluation has revealed that GPMs resulting from partitioned inference algorithms, i.e. 3CS, LARGe, and LGI, deliver similar model quality in comparison to those models produced by non-partitioned algorithms. In addition, runtime of the inference process is reduced considerably especially for larger time series, where partitioned approaches achieve a speed-up factor of up to 50 with regards to non-partitioned methods^7^, i.e. CKS, ABCD and SKC.

## Data Availability

The data, that has been used for the evaluation conducted for this paper, is available via the sources cited in Table 2.

## Footnotes

1 In contrast to CKS and ABCD, SKC also includes a so-called kernel buffer. To increase comparability with CKS and ABCD, we abstract from that kernel buffer by using buffer size 1, which is often used by Kim and Teh [25] themselves.

2 Focusing on uni-dimensional input data, such as time series, LARGe has also been called Timeseries Automatic GPM Retrieval (TAGR) in an earlier publication [7].

3 A mechanism to return a preliminary GPM is not included in other automatic GPM inference algorithms. To increase comparability, this mechanism is omitted in our evaluations of LARGe as well. In doing so, line 5 of Algorithm 2 is changed to $i = min(\mathcal {I})$ corresponding to the *sequential traversal strategy* presented in [6].

4 To increase comparability, we use the same complexity constraint for 3CS as for the other algorithms. Originally, 3CS was proposed accompanied by a slightly different constraint [9].

5 In Fig. 1, we use the measure of relative runtime. It is defined as the original measure divided by the average one for the respective dataset across all algorithms. In doing so, it abstract from the peculiarities of a dataset, such as dataset size and its complexity.

6 Analogously to Figs. 1, 2 illustrates model quality via the relative RMSE.

7 Fastest non-partitioned approach on the largest dataset, where execution was not aborted, is SKC on uni-dimensional *Power Plant*. SKC needed about 20,305 seconds (i.e. 05:38:25), while LGI terminated after 360 seconds (i.e. 00:06:00): $\frac{20,305}{360} \approx 56.$

## Code availability

The code, that has been used for the evaluation conducted for this paper, is available via our own GitHub repository(cf. https://github.com/Bernsai/GaussianProcessModelRetrieval) dedicated to model inference algorithms for Gaussian Processes. It encompasses an implementation for all the evaluated algorithms.
